# Exposure to Mitochondrial Genotoxins and Dopaminergic Neurodegeneration in *Caenorhabditis elegans*

**DOI:** 10.1371/journal.pone.0114459

**Published:** 2014-12-08

**Authors:** Claudia P. González-Hunt, Maxwell C. K. Leung, Rakesh K. Bodhicharla, Madeline G. McKeever, Andrew E. Arrant, Kathleen M. Margillo, Ian T. Ryde, Derek D. Cyr, Sara G. Kosmaczewski, Marc Hammarlund, Joel N. Meyer

**Affiliations:** 1 Nicholas School of the Environment, Duke University, Durham, North Carolina, United States of America; 2 Department of Pharmacology and Cancer Biology, Duke University, Durham, North Carolina, United States of America; 3 Center for Applied Genomics and Technology, Duke University, Durham, North Carolina, United States of America; 4 Department of Genetics, Program in Cellular Neuroscience, Neurodegeneration, and Repair, Yale University School of Medicine, New Haven, Connecticut, United States of America; National Institute of Health, United States of America

## Abstract

Neurodegeneration has been correlated with mitochondrial DNA (mtDNA) damage and exposure to environmental toxins, but causation is unclear. We investigated the ability of several known environmental genotoxins and neurotoxins to cause mtDNA damage, mtDNA depletion, and neurodegeneration in *Caenorhabditis elegans*. We found that paraquat, cadmium chloride and aflatoxin B_1_ caused more mitochondrial than nuclear DNA damage, and paraquat and aflatoxin B_1_ also caused dopaminergic neurodegeneration. 6-hydroxydopamine (6-OHDA) caused similar levels of mitochondrial and nuclear DNA damage. To further test whether the neurodegeneration could be attributed to the observed mtDNA damage, *C. elegans* were exposed to repeated low-dose ultraviolet C radiation (UVC) that resulted in persistent mtDNA damage; this exposure also resulted in dopaminergic neurodegeneration. Damage to GABAergic neurons and pharyngeal muscle cells was not detected. We also found that fasting at the first larval stage was protective in dopaminergic neurons against 6-OHDA-induced neurodegeneration. Finally, we found that dopaminergic neurons in *C. elegans* are capable of regeneration after laser surgery. Our findings are consistent with a causal role for mitochondrial DNA damage in neurodegeneration, but also support non mtDNA-mediated mechanisms.

## Introduction

The possible mitochondrial toxicity of environmental pollutants has attracted increasing interest in recent years [1]–[4], and mitochondrial DNA (mtDNA) may represent a particularly critical target. The human health significance of the mitochondrial genome as a target of genotoxins has recently received greater attention [5]–[10]. mtDNA is potentially more prone to damage than nuclear DNA (nDNA) due to the tendency of lipophilic and certain charged chemicals to accumulate in the mitochondria [11]–[14], the proximity of mtDNA to electron transport chain-mediated production of reactive oxygen species (ROS), and the absence of chromatin packing and many DNA repair pathways [15]–[17]. Furthermore, non-genotoxic mitochondrial toxins may indirectly cause mtDNA damage by disrupting oxidative phosphorylation [18]–[20]. A number of genotoxins cause more mtDNA than nDNA damage [21]–[24] but the opposite is true for other chemicals [23], [25], [26], and relatively few chemicals have been tested for their potency in damaging the mitochondrial genome (reviewed in Meyer et al. [4]).

Mitochondria play an important role in multiple neurological disorders [18], [27]. Neurons are high energy-use cells that rely on mitochondria for their supply of energy [6]. The high metabolic activity of neurons leads to the production of ROS, and the brain is particularly susceptible to oxidative stress due to its low supply of antioxidant enzymes and high lipid content [28], [29]. mtDNA damage and mutation have been correlated with neurodegeneration [30]–[37]. A recent study showed dopaminergic neurodegeneration in mice exhibiting mtDNA doublestrand breaks produced by a mitochondrial-targeted restriction enzyme [38]. Another recent study detected oxidative mtDNA lesions in the brain of Parkinson's disease (PD) patients, and also *in vivo* and *in vitro* after mitochondrial impairment by rotenone [39]. Furthermore, mutations in the only mtDNA polymerase, DNA polymerase γ, can result in parkinsonism in humans [40], [41].

A significant portion of neurodegenerative disease, especially PD, may result from environmental exposures [42]. Epidemiological studies have identified an association between neurodegeneration and exposure to environmental chemicals including pesticides and heavy metals [43]–[48], and laboratory studies support the ability of some of these chemicals to cause neurodegeneration [49], [50]. These chemicals, however, have not been tested for their relative genotoxicity in the nuclear and mitochondrial genomes.

Finally, there is growing evidence that neurodegeneration can result from early lifestage exposures [51]–[53]. Environmental genotoxins that target the mtDNA are strong candidates for acting in this fashion. Since mtDNA copies in somatic cells are all amplified from a smaller pool of mtDNA in the embryo [54], the mtDNA damage resulting from environmental exposure in early life stages may impact physiological functions in a later stage of life.

Thus, mtDNA is particularly vulnerable to many environmental pollutants, mtDNA damage can cause neurodegeneration, and some neurodegenerative diseases are associated with exposure to environmental chemicals. These associations suggest the possibility that environmental pollutants that cause mtDNA damage (i.e., “environmental mito-genotoxins”) could also cause neurodegeneration.

We carried out a series of experiments to examine whether or not (a) important environmental genotoxins and neurotoxins could cause mtDNA damage or depletion, (b) mitochondrial genotoxins could cause dopaminergic neurodegeneration, and if (c) the observed dopaminergic neurodegeneration could be attributed specifically to mtDNA damage. The chemicals that we tested include chemicals associated in the experimental and/or epidemiological literature with PD (paraquat, rotenone, maneb, and manganese) as well as chemicals that are known genotoxins and mitochondrial poisons (aflatoxin B_1_ and cadmium). We utilized 6-OHDA as a positive control for chemical-induced dopaminergic neurodegeneration [55].

Our findings support a potential causal role for mtDNA damage resulting from exposure to environmental chemicals in neurodegeneration. Our experiments also led us to the observations that fasting early in life was protective against 6-OHDA-induced dopaminergic neurodegeneration, and that dopaminergic neurons in *C. elegans* are capable of regeneration.

## Materials and Methods

### *C. elegans* culture

Populations of *C. elegans* were maintained on K agar plates [56] seeded with OP50 bacteria. Synchronized populations of nematodes were obtained by bleach-sodium hydroxide isolation of eggs. L1 growth-arrested (starved) larvae were obtained by hatching eggs in K^+^ medium, previously referred to as “complete K-medium” [57]. All transfers were made by washing nematodes off of agar plates and rinsing (after centrifugation at 2200 *g* for 2 min) in K medium.

The germline-deficient JK1107 strain (*glp-1*) of *C. elegans* was obtained from the *Caenorhabditis* Genetics Center (University of Minnesota). The transgenic strains BY250 (*vtIs7[Pdat-1::GFP]*, expressing GFP only in dopaminergic neurons) and CZ1200 (*unc-25::GFP*, expressing GFP only in GABAergic neurons) were generously provided by Michael Aschner (Vanderbilt University). Strain XE1311 *(vtIs7[Pdat-1::GFP];mkk-4(ju91))* was generated by crossing the *mkk-4(jk91)* mutation into the *vtIs7* background.

### Chemical exposures

Aflatoxin B_1_ (AFB_1_), paraquat, rotenone, maneb, manganese chloride (MnCl_2_), cadmium chloride (CdCl_2_) and 6-OHDA HCl were purchased from Sigma (St Louis, MO). Paraquat, MnCl_2_, and CdCl_2_ were dissolved into K^+^ medium for L1 larvae or K medium for young adults. 6-OHDA was dissolved into a solution of ascorbic acid in K^+^ medium. AFB_1_, rotenone, and maneb were dissolved in dimethyl sulfoxide (DMSO) to prepare stock solutions (100x the final treatment concentration). *C. elegans* were treated with the solutions in 12-well plates. Stock solutions were added to treatment wells at a maximum amount of 1 % (v/v) in K^+^ or K medium. Each well contained 1 ml of the treatment solution, 1000 L1 larvae, or 300 adults, and OP50 in the case of the adults. 1% DMSO did not affect nematode growth or survival (data not shown).

### Adult lethality assay

The acute toxicities of paraquat, rotenone, maneb, MnCl_2_, and CdCl_2_ were determined in *glp-1* nematodes prior to the DNA damage assay. Synchronized *glp-1* nematodes used in this assay were grown at 15°C for 40 h and sterilized at 25°C for 18 h. The worms were then exposed to AFB_1_, paraquat, rotenone, maneb, MnCl_2_, and CdCl_2_ for 24 h. 50 animals were examined in two separate experiments with lethality defined as a lack of response to gentle probing with platinum wire. AFB_1_ was previously found to cause no acute lethality in adult *C. elegans* to its solubility limit (100 µM) [58].

### DNA damage assay

Synchronized *glp-1* worms, grown at 15°C for 40 h and sterilized for 18 h at 25°C, were exposed to 3, 30 and 100 µM AFB_1_, 0.6, 6 and 20 mM paraquat, 0.6, 6 and 21 µM rotenone, 23, 226 and 754 µM maneb, 0.75, 7.5, and 25 mM MnCl_2_, and 0.03, 0.3 and 1 mM CdCl_2_ in K medium in the presence of OP50 for 48 h. The highest exposure level chosen for each chemical was the one that resulted in no or minimal lethality, as observed in our 24 h adult lethality assay. No mortality was observed during the 48 h exposures. The solubility limit was chosen if lethality was not detected. The worms were also exposed to 50, 100 mM and 150 mM 6-OHDA. 6-OHDA exposures were constrained to 1 h due to the short half-life of 6-OHDA in aqueous solution, and no mortality was observed during this exposure. Six worms were picked and pooled in a single tube per biological replicate, and eight biological replicates were taken per treatment (three replicates for 6-OHDA) in two experiments separated in time.

nDNA and mtDNA damage were evaluated using a QPCR-based method as previously described [59], [60]. This assay defines the control samples as undamaged and determines a lesion frequency in experimental samples based on any decrease in amplification efficiency relative to the control samples [16]. Two nuclear genome targets (*unc-2* and small nuclear; 9.3 and 0.2 kb) and two mitochondrial genome targets (10.9 and 0.2 kb) were amplified. The amount of long PCR product provides a measurement of lesion frequency, while the amount of short PCR product provides normalization to DNA concentration as well as a relative measure of mitochondrial genome copy number [59].

Utilizing a germline-deficient mutant (*glp-1*) for this experiment allowed us to study DNA damage in adults composed entirely of non-dividing cells. Young adult *C. elegans* have a rapidly proliferating germ line, so that DNA damage caused by chemical exposure could be readily “diluted” by the new DNA produced by dividing germ cells, reducing the apparent level of DNA damage [61]. The confounding effect of DNA replication can be minimized by using a *glp-1* mutant strain since outside of the germ line, no cell divisions occur in adult *C. elegans* [62].

### L1 development assay

The growth inhibitory effects of DMSO, AFB_1_, CdCl_2_, and paraquat were determined on L1 larvae prior to neurodegeneration studies. 500 L1 larvae were exposed to DMSO, AFB_1_, CdCl_2_, and paraquat for 48 h in the absence of food, and then transferred to seeded K agar plates. Nematodes from the exposed populations were scored every 24 h after the treatment for 3 days, and developmental delay was evaluated in comparison to the untreated control. The experiment was repeated once (i.e. two to four biological replicates in total). The highest concentration that resulted in minimal decrease in size as compared to the control was used as a basis to choose highest exposure level in neurodegeneration studies.

### Dopaminergic and GABAergic neurodegeneration

Synchronized BY250 and CZ1200 L1s were exposed to AFB_1_, paraquat, and CdCl_2_ in K^+^ medium in the absence of OP50 for 48 h. BY250 L1 larvae were exposed to 6-OHDA for 1 h. The worms were washed with K medium twice, transferred to seeded K agar plates, and sampled 24, 48, and 96 h after the exposure.

Treated *C. elegans* were picked onto a 2% agar pad and immobilized with 15 µl of 1% sodium azide (Sigma-Aldrich). Nematodes were examined using a Zeiss Axioskop microscope and neuronal morphology was assessed by individual observation of each cephalic (CEP) neuron. Neurons were assigned a score from 0 to 2 based on the amount of morphological abnormalities present. Each experiment was repeated three or four times with ∼100 worms analyzed per treatment at each time point. For the 6-OHDA exposures, 10–15 worms were analyzed per treatment for each time point, and the experiment was repeated twice. CZ1200 worms were analyzed similarly, focusing on damage to the ventral motor neural cord. All scoring was double-blinded.

### UVC and ethidium bromide treatment

*C. elegans* were exposed to serial UVC doses over 48 h as described in Bess et al. [63]. This exposure protocol is based on the fact that UVC-induced DNA damage is quickly repaired in the nuclear but not mitochondrial genome [61], resulting in accumulation of mtDNA damage while permitting repair of nDNA [63], [64]. This protocol results in larval growth delay that is mild at the doses used in this study [63]. Ethidium bromide exposures were performed on K agar plates [65] using doses leading to mild larval growth delay [63].

### Laser surgery

The dendrites of the CEP neurons were severed with a pulsed laser essentially as described [66]. L4 stage BY250 *vtIs7[Pdat-1::GFP]* and XE1311 *vtIs7[Pdat-1::GFP];mkk-4(ju91)* worms were used. 2 CEP neurons were cut in each worm, at the point just anterior to the curve made around the anterior pharyngeal bulb. Worms were recovered onto NGM plates at 20°C for 24 h before being remounted and scored for regeneration. Regeneration was defined as anterior growth beyond the cut site. Representative images were collected on a Zeiss LSM710 point scanner and analyzed with ImageJ (U. S. National Institutes of Health, Bethesda, Maryland, USA).

### Statistical analysis

DNA damage and relative mtDNA copy number data were analyzed with Statview for Windows (Version 5.0.1, SAS Institute Inc., Cary, NC) and JMP Pro for Windows (Version 11.0.0, SAS Institute Inc., Cary, NC). One- or two-factor analysis of variance (ANOVA) was used and each chemical was analyzed as an independent experiment. A *p*-value of less than 0.05 was considered statistically significant.

Dopaminergic neurodegeneration data were analyzed using the statistical software R version 2.12.0 (R Foundation for Statistical Computing, Vienna, Austria), Statview or JMP. The nonparametric Kruskal-Wallis test was used to test for differences between dosage levels for each chemical at each time point. Due to sparseness in the cross-tabulations of dosage levels and scores, Fisher's exact test (FET) was used when testing independence of chemical dosage levels and scores at each time point. FET was also used to analyze laser axotomy data. A *p*-value of less than 0.05 was considered statistically significant.

## Results

### Paraquat, AFB_1_ and CdCl_2_ caused more mitochondrial than nuclear DNA damage

**Figure 1:**
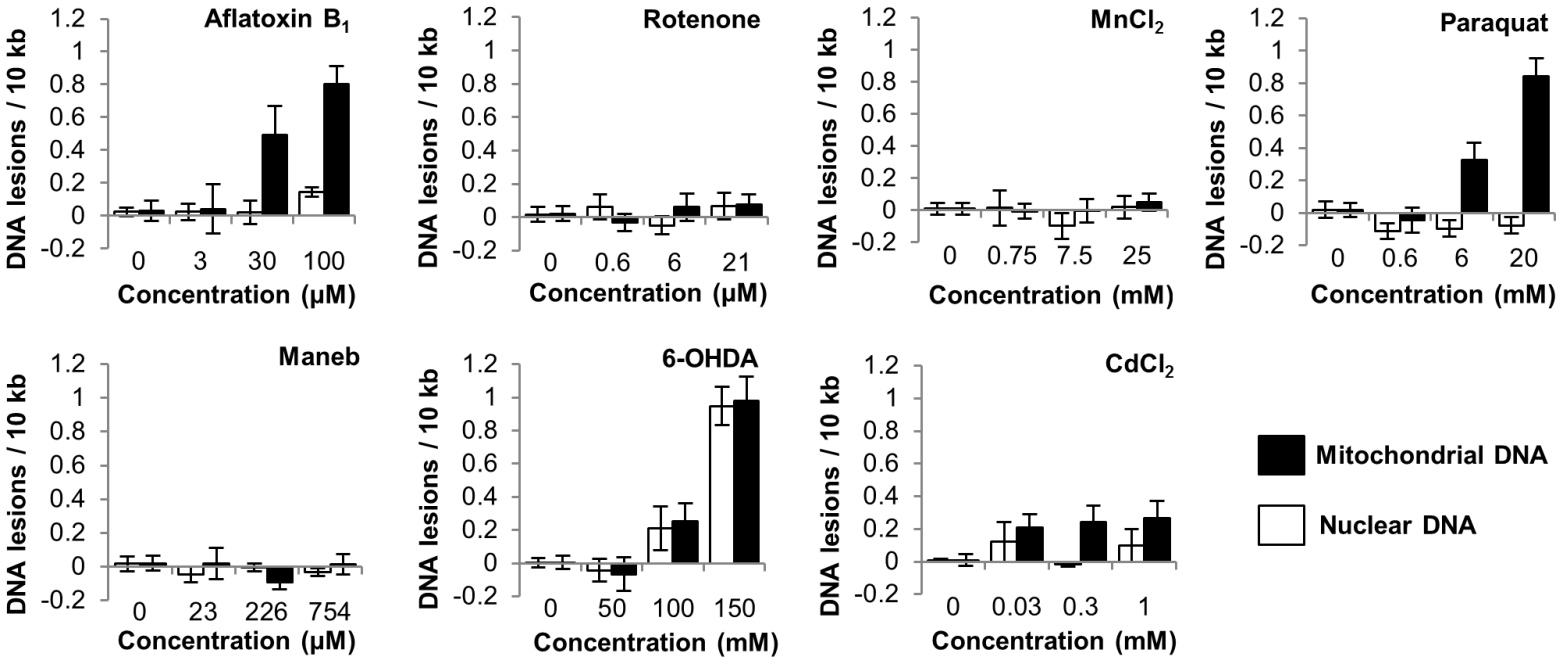
Exposures to aflatoxin B_1_, paraquat, cadmium chloride and 6-OHDA caused detectable DNA damage. p<.0001, p<.0001, p = 0.028, p<.0001 respectively for main effects of dose. Aflatoxin B_1_, paraquat and cadmium chloride caused more damage to the mitochondrial than nuclear genomes (p<.0001, p<.0001, and p = 0.0125 respectively for main effect of genome). Data analyzed by Two-way ANOVA. Bars ± SEM.

We first tested the ability of known neurotoxins or genotoxins to cause either mitochondrial or nuclear DNA damage. The doses used were sublethal (S1 Table), chosen as described in Materials and Methods. Paraquat, AFB_1_, and 6-OHDA exposure resulted in dose-dependent DNA damage (p<0.0001, <0.0001, and <0.0001 respectively, for main effects of dose, Fig. 1) in *C. elegans*. Exposure to CdCl_2_ also resulted in DNA damage (p = 0.028), although this damage was not dose-dependent (p>0.05 for all pairwise comparisons except each dose compared to control). Rotenone, maneb, and MnCl_2_ did not cause detectable DNA damage (p>0.05 for the effect of dose in all cases, Fig. 1). mtDNA was more susceptible than nDNA to damage due to AFB_1_, paraquat or CdCl_2_ exposure (p<0.0001, <0.0001, and p = 0.0125 respectively for main effect of genome).

### MnCl_2_ and CdCl_2_ caused a decrease in mtDNA:nDNA ratio

**Figure 2:**
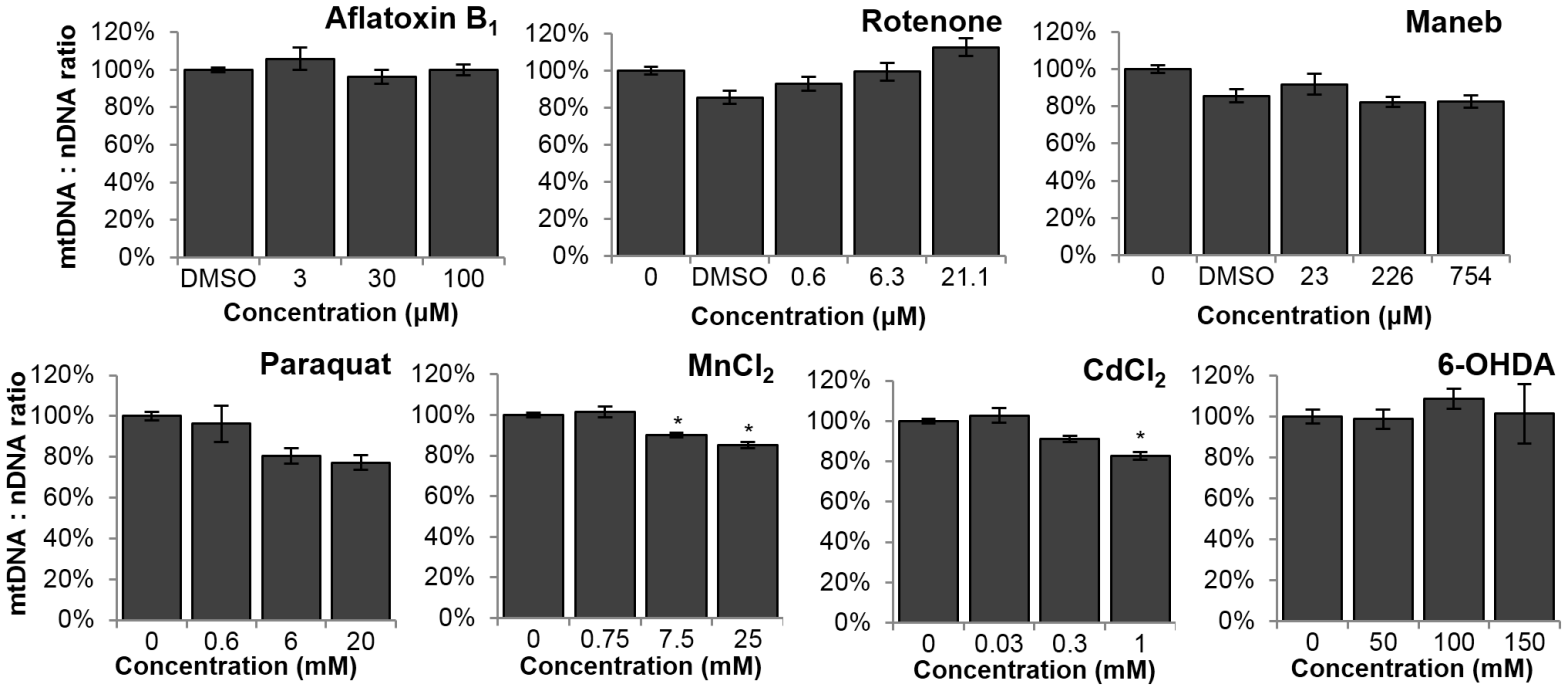
Manganese chloride and cadmium chloride resulted in a decrease in the relative mtDNA:nDNA ratio. Ratio defined as 100% in controls. For MnCl_2_, one-way ANOVA, effect of concentration p = 0.03; Fisher's PLSD, p = 0.0231 for 7.5 mM and p = 0.0015 for 25 mM. For CdCl_2_, one-way ANOVA, effect of concentration p = 0.0091; Fisher's PLSD, p = 0.0032 for 1 mM. Bars ± SEM.

Some environmental exposures are known to result in depletion [67] or proliferation [68] of the mitochondrial genome. To test for such effects, a relative mtDNA:nDNA ratio was calculated by comparing the amount of PCR products amplified from small nDNA and mtDNA targets [59], [69]. The mtDNA:nDNA ratio was defined as 100% in controls. Exposures to MnCl_2_ and CdCl_2_ resulted in a dose-dependent change in the mtDNA:nDNA ratio (Fig. 2. For MnCl_2_, one-way ANOVA, effect of concentration p = 0.03; Fisher's PLSD, p = 0.0231 for 7.5 mM and p = 0.0015 for 25 mM. For CdCl_2_, one-way ANOVA, effect of concentration p = 0.0091; Fisher's PLSD, p = 0.0032 for 1 mM).

### Establishment of a positive control-based scoring system for dopaminergic neurodegeneration

**Figure 3:**
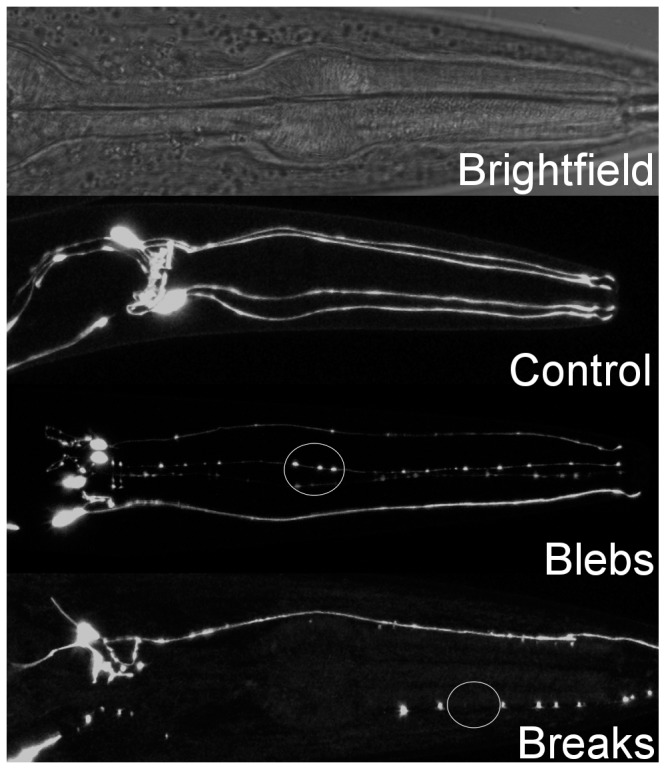
Representation of progressive damage in dopaminergic neurons after 6-OHDA exposure. The circled areas include typically observed abnormalities, referred to as blebs and breaks in this publication.

6-OHDA is a known selective dopaminergic neurodegenerative agent. We carried out dose-response studies (S1 Figure, Fig. 3) to establish a scoring system for dopaminergic neurodegeneration. GFP-tagged CEP neurons were observed and assigned a numerical score based on neuronal morphology. The scoring system is as follows: 0 =  No observable damage, 0.5 =  Dendrite has <5 blebs, no observable breaks, 1 =  Dendrite has ≥5 blebs, <5 breaks, 1.5 =  Dendrite has ≥5 blebs and ≥5 breaks, 2 =  Dendrite shows same amount of damage as in a score of 1.5, plus the neuronal cell body is rounded, 2.5 =  Neuron structure is unrecognizable. Interestingly, the four CEP neurons damaged in an individual often responded very differently, with affected individuals frequently displaying a combination of highly damaged and entirely intact morphologies.

### AFB_1_ and paraquat exposures result in dopaminergic neurodegeneration

We next tested the hypothesis that chemical exposures causing mtDNA damage can also result in neurodegeneration in *C. elegans*. The doses used were the highest doses that resulted in no or minimal growth inhibition as determined in the L1 development assay (S2 Table). We detected a dose-dependent increase in lesions in dopaminergic CEP neurons after paraquat and AFB_1_ exposure, but no damage after CdCl_2_ exposure (Table 1, S2 Figure). Although the 24 h *p* value for CdCl_2_ is borderline statistically significant, the biological effect is very small if it is real. We only assessed neurodegeneration in chemicals that caused mtDNA damage in our damage assay, but it is important to mention that manganese, maneb, and rotenone have already been shown to cause dopaminergic neurodegeneration in *C. elegans* [70]–[74]. Neither significant lesions in the GABAergic dorsal nerve cord nor any sign of pharyngeal necrosis (measured as described in Samokhvalov et al. [75]) were detected (data not shown). Interestingly, the amount of damage detected in paraquat- and AFB_1_-treated animals was lower at 96 than 48 h (this was not the result of death of more-injured individuals, since there was no lethality).

**Table 1:** Dopaminergic neurodegeneration caused by exposure to paraquat, aflatoxin B1 and cadmium chloride in early stage *C. elegans*.

Chemical and Time after Exposure		Concentration and Damage Score (Sample Size)			
**Paraquat**		**0 µM**	**54 µM**	**180 µM**	***p*** ** value**
	**24 hr**	0.13 (124)	0.16 (84)	0.33 (82)	0.050*
	**48 hr**	0.01 (135)	0.05 (87)	0.10 (67)	0.007*
	**96 hr**	0.03 (172)	0.04 (94)	0.03 (95)	0.864
**Aflatoxin B_1_**		**0 µM**	**30 µM**	**100 µM**	***p*** ** value**
	**24 hr**	0.10 (83)	0.17 (75)	0.26 (100)	0.002*
	**48 hr**	0.07 (94)	0.07 (75)	0.10 (93)	0.098
	**96 hr**	0.07 (93)	0.06 (93)	0.06 (95)	0.717
**Cadmium chloride**		**0 µM**	**240 µM**	**800 µM**	***p*** ** value**
	**24 hr**	0 (45)	0 (45)	0.01 (45)	0.0492*
	**48 hr**	0 (45)	0 (45)	0 (45)	1.000
	**96 hr**	0 (45)	0 (45)	0 (45)	1.000

Neuronal damage was scored from 0 (lowest) to 2.5 (highest) and assessed statistically using the Kruskal-Wallis test. Number of worms scored in parentheses, asterisks (*) denote statistical significance.

### Fasting is protective in the context of chemical-induced dopaminergic neurodegeneration in *C. elegans*

**Figure 4:**
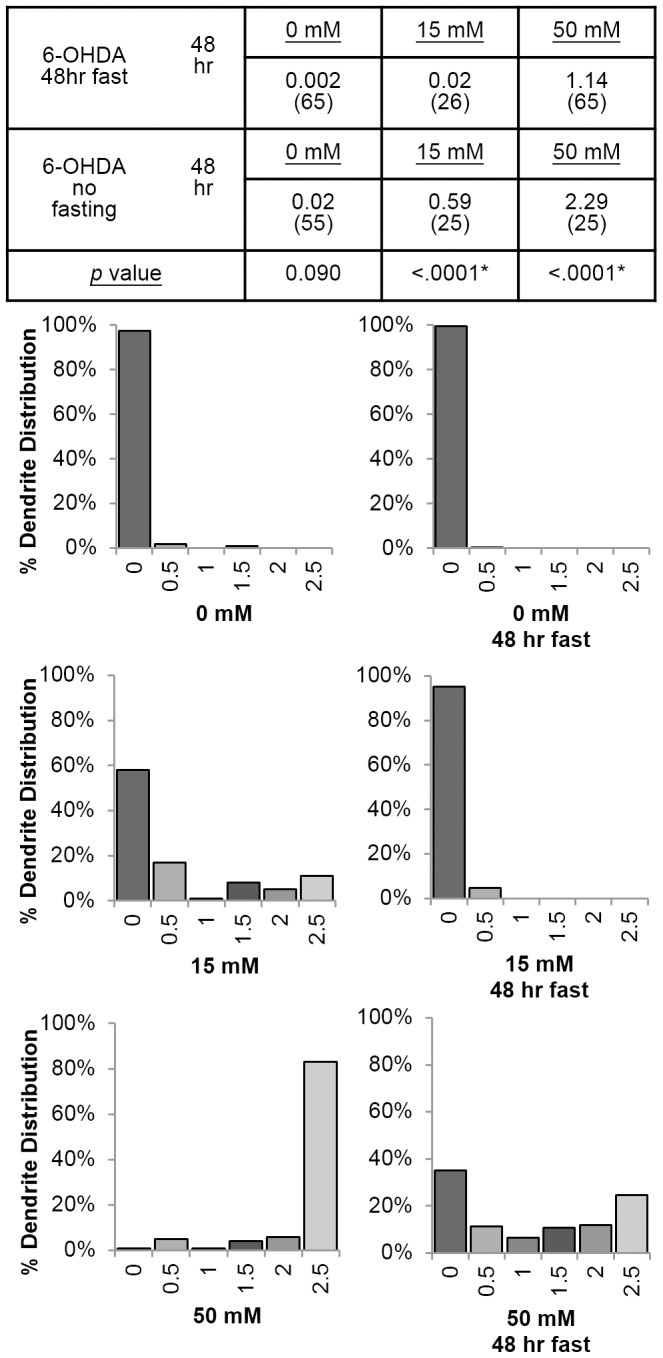
Fasting protects against 6-OHDA–induced dopaminergic neurodegeneration. Neuronal damage was scored from 0 (lowest) to 2.5 (highest) and assessed statistically using the Fisher's Exact Test.

Since we exposed L1 nematodes to chemicals for a 48 h period without food, and caloric restriction is known to increase stress resistance in some contexts [76]–[78], we tested whether this could have had a neuroprotective effect by assessing neuronal damage in BY250 *C. elegans* exposed to 15 mM and 50 mM 6-OHDA. We found that animals exposed to 6-OHDA after a 48 h period of fasting were significantly more resistant to dopaminergic neurodegeneration than animals exposed immediately after the overnight liquid hatch (Fig. 4); others have made the same observation [79]. This suggests that our chemical neurodegeneration results are conservative compared to what would be observed in nematodes that had not been fasted. To test if starvation was protective by reducing the degree of DNA damage incurred, we repeated our young adult *C. elegans* DNA damage assay after paraquat exposure, but included a 48 h starvation period prior to dosing. We measured slightly (∼25%) less mtDNA damage in starved worms dosed with 6 mM and 20 mM paraquat (S3 Figure), but this was not statistically significant (three-way ANOVA, p = 0.1411 for the interaction between dose, genome, and starvation status). This result suggests that the protective effect of absence of food was mediated by the biological response to damage, rather than by protection against the damage that was initially incurred. We note that DNA damage measurements (Fig. 1) were made in fed, not starved, nematodes.

### Dopaminergic neuron damage can be regenerated in *C. elegans*

**Figure 5:**
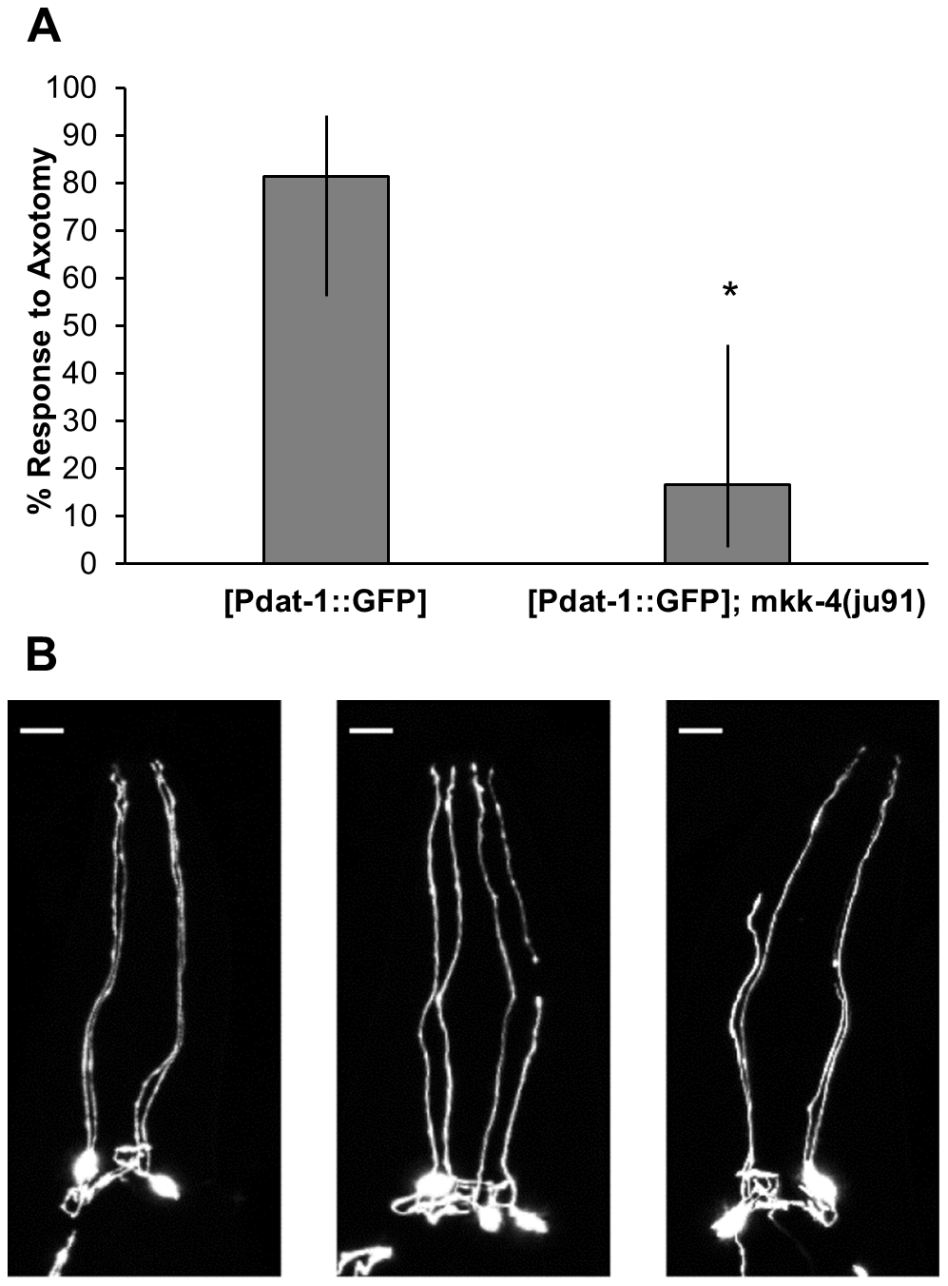
Damage to dopaminergic neurons caused by laser ablation is repaired. A, After laser ablation, 81% of vtIs7[Pdat-1::GFP] worms showed neuronal anterior growth, compared to 17% of the vtIs7[Pdat-1::GFP];mkk-4(ju91) worms tested (p = 0.0016, Fisher's Exact Test, bars represent 95% CI). B, From left to right, images are: uncut (with dashed line representing cut site), no response, and response. Scale bars are 10 µm.

Damage to the dopamine neurons after paraquat and AFB_1_ exposure decreased over time, from a peak at 24 hours down to undetectable levels at 96 hours post-exposure (Table 1). One possible explanation for this decrease is that regenerative processes restore normal morphology to injured neurons. However, the regenerative potential of the CEP dendrites has not been determined. To assess whether these neurons are capable of regenerating after damage, we tested their ability to respond to pulsed laser surgery. Many neurons in *C. elegans* can respond to laser surgery by initiating regeneration [80] but the dendrites of the dopamine neurons have not been tested. We found that the CEP dendrites are capable of injury-induced growth after laser surgery: 13 out of 16 severed dendrites exhibited anterior growth beyond the cut site (Fig. 5). Next, we asked whether injury-induced growth of the CEP dendrites requires the critical *dlk-1* MAP kinase pathway, which is important for regeneration in a variety of neuron types in *C. elegans* [81], [82]. We found that animals defective for *dlk-1* pathway signaling (*mkk-4* mutants) have reduced CEP regeneration (Fig. 5), suggesting that regeneration of the CEP dendrite has some molecular similarities to axon regeneration.

We then explored the effect of the *dlk-1* MAP kinase pathway in the response to chemical-induced neurodegeneration, as chemically induced neuronal damage could be different from that caused by laser surgery and therefore trigger a different regenerative response. We tested the effect of the *mkk-4* mutation on the response to 6-OHDA, as this chemical causes the widest range of neuronal damage scores. The XE1311 worms (*mkk-4* KO) were more sensitive to 6-OHDA than wild type BY250 at almost every time point and dose as determined by the Fisher's exact test (S4 Figure). However, we did not observe regeneration in the BY250 worms; damage worsened over time for both strains of *C. elegans*. These results suggest that the MAP kinase pathway response to neuronal damage depends on the type of damage induced, as the responses we saw to chemical injury and laser surgery were different. Furthermore, exposure to different chemicals may or may not trigger regenerative pathways in response to neuronal damage, with damage caused by some toxins (paraquat and AFB_1_) but not others (6-OHDA) exhibiting apparent regeneration over time.

Together, these data suggest that recovery of CEP dendrite morphology after paraquat and AFB_1_ exposure could be mediated by injury response pathways, possibly involving the conserved *dlk-1* MAP kinase pathway.

### UVC and ethidium bromide exposures result in dopaminergic neurodegeneration

Although paraquat, AFB_1_, and CdCl_2_ caused more mtDNA than nDNA damage, we could not rule out that the nDNA damage and other forms of macromolecular damage caused by these chemicals might also have caused the observed neurodegeneration. To further explore the possibility that mtDNA damage on its own could lead to dopaminergic neurodegeneration we exposed *C. elegans* to (a) repeated low-dose UVC radiation that results in persistent DNA damage in the mitochondria but not in the nucleus [63], [64]; and/or (b) ethidium bromide that blocks mtDNA replication [83] and exacerbates some effects of persistent mtDNA damage in this model organism, but not the induction of DNA damage per se [63], [64]. We note that we did not employ UVC and ethidium bromide because of exposure relevance in the context of PD (UVC does not reach dopaminergic neurons in people, and ethidium bromide is not a relevant chemical from the perspective of human neurodegeneration), but rather as mechanistic tools.

Exposures to both UVC and combined UVC and ethidium bromide treatment resulted in neuronal lesions 48 and 96 h after the exposure (Table 2, S2 Figure). Exposure to ethidium bromide alone resulted in a low level of lesions, and only at one time, 48 h after the exposure (Table 2). The combined UVC and ethidium bromide treatment did not result in a higher level of lesions as compared to the UVC or ethidium bromide treatment alone.

**Table 2:** Dopaminergic neurodegeneration caused by exposure to ultraviolet C radiation, ethidium bromide, and both in early stage *C. elegans*.

Chemical and Time after Exposure		Concentration and Damage Score (Sample Size)			
**Ultraviolet C radiation (UVC)**		**0 J/m^2^**	**10 J/m^2^**		***p*** ** value**
	**24 hr**	0.13 (124)	0.23 (96)		0.137
	**48 hr**	0.01 (135)	0.27 (139)		<0.001*
	**96 hr**	0.03 (172)	0.11 (135)		0.003*
**Ethidium bromide (EtBr)**		**0 µM**	**5 µM**	**10 µM**	***p*** ** value**
	**24 hr**	0.13 (124)	0.16 (83)	0.19 (118)	0.414
	**48 hr**	0.01 (135)	0.08 (96)	0.11 (130)	0.011*
	**96 hr**	0.03 (172)	0.02 (95)	0.07 (172)	0.082
**UVC + EtBr**		**0 µM**	**5 µM+5 J/m^2^**	**5 µM+10 J/m^2^**	***p*** ** value**
	**24 hr**	0.13 (124)	0.26 (116)	0.16 (104)	0.082
	**48 hr**	0.01 (135)	0.07 (130)	0.16 (123)	<0.001*
	**96 hr**	0.03 (172)	0.07 (157)	0.19 (167)	<0.001*

Neuronal damage was scored from 0 (lowest) to 2.5 (highest) and assessed statistically using the Kruskal-Wallis test. Number of worms scored in parentheses, asterisks (*) denote statistical significance.

## Discussion

Our results show that the important environmental pollutants paraquat, AFB_1_ and CdCl_2_ can cause more mtDNA than nDNA damage in the nematode *C. elegans*. Early developmental exposure to paraquat and AFB_1_ also caused dopaminergic neurodegeneration. Our experiments also showed that fasting can have a neuroprotective effect, and that the dendrites of dopaminergic CEP neurons can regenerate in this model organism. It will be important to consider the possibility of regeneration, and effects of starvation, when interpreting results from chemical exposures and neuronal damage assessment assays performed with this model organism.

We report for the first time that paraquat, a heavily used herbicide, selectively targets mtDNA over nDNA. This could be due to the fact that paraquat is a potent redox cycler, readily accepting electrons and generating superoxide anions [84], [85] and several studies have shown that mtDNA is three to five times more susceptible than nDNA to oxidative damage caused by hydrogen peroxide, organic peroxide, and peroxynitrite [21], [86]–[89]. Exposure to paraquat also disrupts mitochondrial electron transport chain function [18], increases mitochondrial production of ROS [90], [91], and increases oxidative DNA damage in mammalian species *in vivo* [92], [93].

We also found that AFB_1_, a fungal genotoxin widely found in different agricultural products, selectively targets mtDNA in *C. elegans*. This finding is consistent with previous work by Niranjan and colleagues [94], [95] who identified a higher level of AFB_1_ bound to hepatic mtDNA than nDNA in several rodent species. The relative susceptibility of mtDNA versus nDNA was within the same order of magnitude in all these studies. Mitochondrial effects of AFB_1_ are well documented and include alteration of mitochondrial morphology, disruption of respiratory function, and reduction of energy production [96]–[98]. However, AFB_1_ is best known as a nDNA mutagen and hepatic carcinogen [3], [99]. The clinical significance of the mitochondrial genotoxicity of AFB_1_ intoxication is not well understood, as is the case for many other mitochondrial toxins [2], [4].

Paraquat and AFB_1_ exposures result in different types of DNA damage, which are repaired by different repair mechanisms. Paraquat exposure generates oxidative DNA damage [100], much of which is known to be repaired by base excision repair in both the nucleus and mitochondria [15]. Interestingly, 1-methyl-4-phenyl-1,2,3,6-tetrahydropyridine (MPTP), another chemical that causes production of ROS and dopaminergic neurodegeneration, also causes more mtDNA than nDNA damage [101]. AFB_1_ exposure, in contrast, generates bulky DNA adducts [3] which are repaired by nucleotide excision repair, a repair mechanism that has not been identified in mitochondria [102]. Thus, AFB_1_ adducts are expected to be highly persistent in mtDNA. Interestingly, there is a subset of oxidative DNA damage that is probably repaired by nucleotide excision repair [103], and therefore also likely to be persistent in mtDNA.

Cadmium, a heavy metal of considerable occupational and environmental concern, is a known carcinogen that induces DNA damage through multiple mechanisms, including alteration of gene expression, inhibition of DNA repair, and induction of oxidative stress [104]. Cadmium accumulates in mitochondria and disrupts the functions of the organelles through a variety of targets [105], [106]. Our findings show that cadmium causes greater levels of mtDNA than nDNA damage.

We also report that 6-OHDA causes detectable levels of DNA damage *in vivo*. There are several reports in the literature suggesting the presence of damage to DNA after 6-OHDA exposure [107]–[109]. Here we present, to our knowledge for the first time, direct evidence of DNA lesions in both mitochondrial and nuclear genomes after *in vivo* 6-OHDA exposure, further strengthening the association between oxidative DNA damage and neurodegeneration.

Paraquat, AFB_1_, UVC, and the UVC + ethidium bromide combination all result in more mtDNA than nDNA damage, as well as dopaminergic neurodegeneration. However, rotenone and maneb did not result in detectable mtDNA damage despite being known dopaminergic neurotoxins. It may be that such damage occurred, but at later timepoints, at levels that our assay could not detect, or in a subset of cells (e.g. neurons) such that the damage signal was diluted because our assay only detects damage in whole-organism extracts. It is also worth noting that different *C. elegans* strains including *glp-1* [64], [110], [111] can have different responses to the same exposure conditions, so performing these experiments in the *glp-1* background rather than wild type could in principle have influenced the level of DNA damage detected. However, we have previously observed similar levels of UVC-induced DNA damage in N2 and *glp-1* nematodes [61], as well as similar rates of nDNA repair [61]. It seems unlikely that exposure to a complex I inhibitor would not lead to production of ROS and some level of mtDNA damage [20]. Zhou et al. [74] recently reported that exposure to rotenone and Mn led to decreases in mtDNA content; we observed the same after Mn exposure, but not rotenone exposure (Fig. 2). Overall, these results suggest that some mitochondrial genotoxins can also cause neurodegeneration. Whether or not the genotoxicity is directly responsible for the neurodegeneration cannot be determined from our experiments. Furthermore, other toxins capable of causing neurodegeneration did not cause mtDNA damage, suggesting a different mechanism of neurotoxicity. These results are consistent with the idea that PD results from a combination of multiple different factors [112].

We found that dopaminergic neurons were more susceptible than GABAergic neurons and pharyngeal muscle cells to chemical-induced degeneration, although both of those cell types are presumably also dependent on mitochondrial function. The reason for this difference is unclear. Dopamine readily oxidizes to react with proteins, lipids, and nucleic acids and produces neurotoxic derivatives, including 6-OHDA [113], which in turn leads to greater ROS formation. We hypothesize that for mitochondrial genotoxins that cause dopaminergic neuron damage, mtDNA damage and depletion may synergize with the endogenous toxicity of dopaminergic metabolism and result in selective dopaminergic lesions.

We carried out our exposures during early larval stages because we hypothesize that mtDNA is likely to be a particularly susceptible target in early stages of life. Early life stages of mammals exhibit a “bottleneck” characterized by a much lower mtDNA copy number [114], and this is likely true in *C. elegans* as well: although germ cell-specific mtDNA copy numbers have not been reported in *C. elegans*, mtDNA copy number per cell is high in freshly laid eggs, decreases on average (across all cells) during development, and is then increased again in newly produced oocytes [64], [115], [116]. The presence of a bottleneck means that early life-stage mtDNA damage may be converted to mutations and amplified during mtDNA replication [117], [118]. Furthermore, some types of mtDNA damage may also inhibit mitochondrial transcription and replication. All of these effects could lead to mitochondrial dysfunction [63], [64], which is likely to be especially problematic in cells highly dependent on mitochondrial function such as neurons. This is also consistent with the neurodevelopmental basis of PD in which dopaminergic neurons sustain damage in early life stage, resulting in clinical symptoms while their number drops below a critical limit as a part of the aging process [51].

The neurodegeneration that we observed is likely ameliorated in our experimental conditions by the protective effects of fasting and by the ability of *C. elegans* to regenerate damaged dopaminergic neurons. Numerous studies have established a link between caloric restriction and protection from oxidative stress, a likely mechanism of neurotoxicity for many relevant environmental toxins [77], [119]. It also has been shown that caloric restriction or intermittent fasting can ameliorate symptoms of induced neurological disease in animal models [120], [121]. These findings are in agreement with our results, since 6-OHDA exerts its toxicity through auto-oxidation and generation of ROS [122], and it is feasible that a 48 h period of fasting could protect from such damage. While previous work on dopaminergic neurodegeneration in *C. elegans* has not typically including a fasted stage, that work has very likely identified less damage than actually occurred, due to regeneration.

Limitations of our study include the fact that we measured DNA damage at the level of the whole organism, rather than specifically in the dopaminergic neurons, because it is unfortunately not currently logistically possible to measure DNA damage only in the eight dopaminergic neurons present in *C. elegans*. However, we note that UVC is likely to penetrate and cause photodimer formation relatively uniformly, so whole-organism measures of DNA damage are likely highly reflective of neuronal levels at least for this stressor. A second limitation is that while Nass et al. [55] showed that *dat-1*::GFP image-based analysis of neurodegeneration was supported by transmission electron microscopy analysis for 6-OHDA, this has not been tested for other environmental stressors (or genetic deficiencies) to our knowledge.

PD has very strong environmental components [46], [47], [112], [123], but it remains unclear which environmental exposures are the most important. The relationship between environmental factors associated epidemiologically with PD [46], [124], [125] and the mechanistic pathways illustrated in controlled experiments [126], [127] is not entirely clear. Our results support the hypothesis that mtDNA damage plays an important role in the development of some cases of PD. These results also suggest an intriguing mechanism-based hypothesis for epidemiology studies to investigate: environmental exposure to mitochondrial genotoxins during early development may predispose to dopaminergic neurodegeneration later in life.

## Supporting Information

S1 Figure **Establishment of a 6-hydroxydopamine-based scoring system for dopaminergic neurodegeneration.** Neuronal damage was scored from 0 (lowest) to 2.5 (highest) and assessed statistically using the Kruskal-Wallis test.
(TIF)

S2 Figure **Representative dopaminergic neuron damage after treatment with 180 µM paraquat, 10 J/m^2^ UVC, and 50 mM 6-hydroxydopamine.** Visualized via confocal microscopy.
(TIF)

S3 Figure **Starvation did not have a protective effect against paraquat exposure.** Worms were dosed to 54 µM and 180 µM paraquat after a 48 hr starvation period.
(TIF)

S4 Figure **A mutation in the** ***mkk-4*** **gene required for neuronal regeneration worsens dopaminergic neurodegeneration in 6-OHDA-exposed nematodes.**
(TIF)

S1 Table **Lethality caused by toxins of interest in young adult** ***C. elegans*** **.** No lethality was detected in both blank and 1% dimethyl sulfoxide (carrier). n = 100 per chemical per dose.
(TIF)

S2 Table **Growth inhibition caused by exposure to paraquat, aflatoxin B_1_ and cadmium chloride in** ***C. elegans*** **.** The development of L1 BY250 (n = 4) was compared to control and scored on a 4 point scale: 1: mostly dead and dying; 2: obvious decrease in size and motility; 3: slight decrease in size and motility; 4: similar to control.
(TIF)
