# Supplementary figures and images for: Exposure to Mitochondrial Genotoxins and Dopaminergic Neurodegeneration in Caenorhabditis elegans
Source: PLoS One. 2014 Dec 8;9(12):e114459. doi: 10.1371/journal.pone.0114459 (PMC4259338; doi:10.1371/journal.pone.0114459)

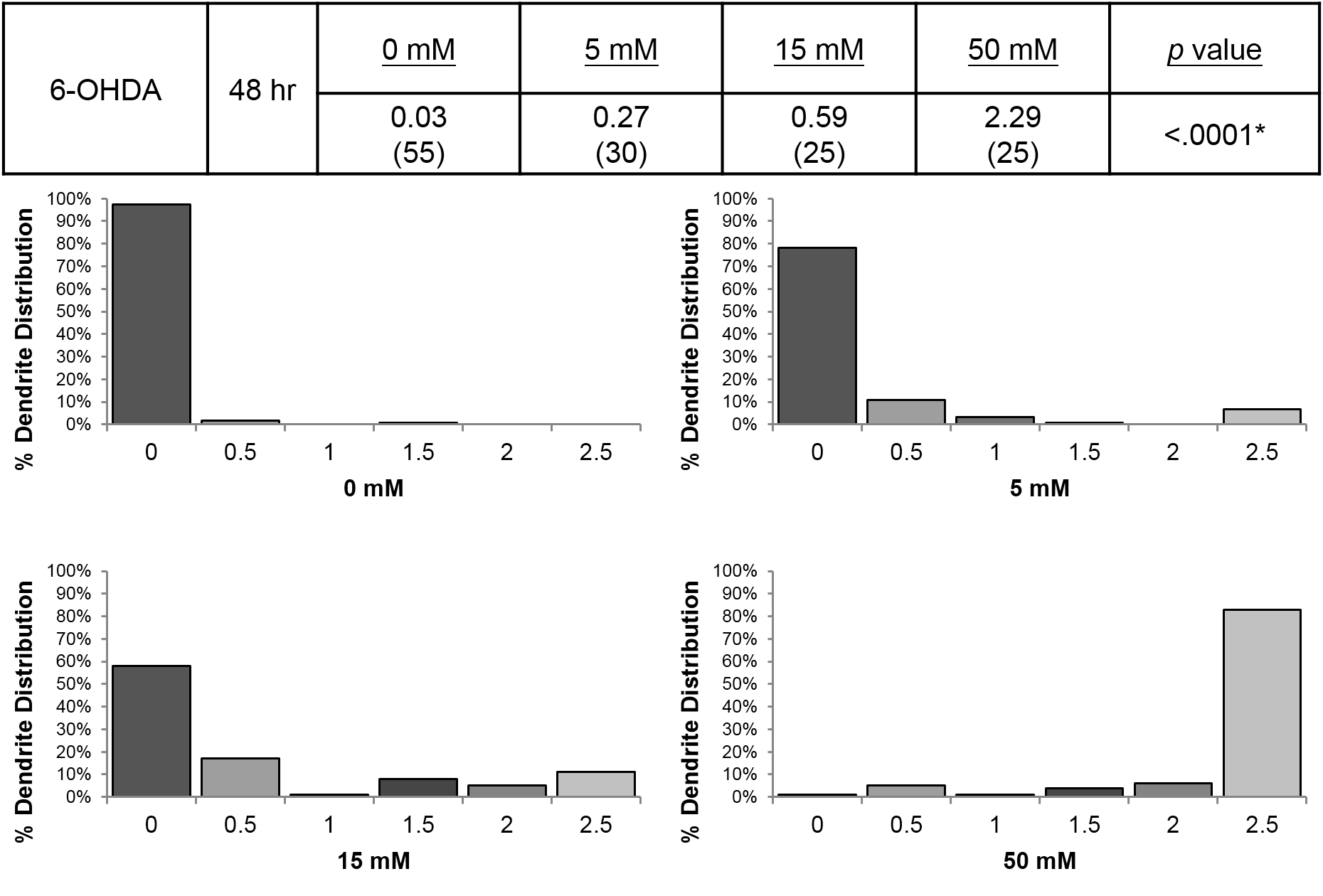

Supplement: S1 Figure — Establishment of a 6-hydroxydopamine-based scoring system for dopaminergic neurodegeneration. Neuronal damage was scored from 0 (lowest) to 2.5 (highest) and assessed statistically using the Kruskal-Wallis test. (TIF) [file pone.0114459.s001.tif]

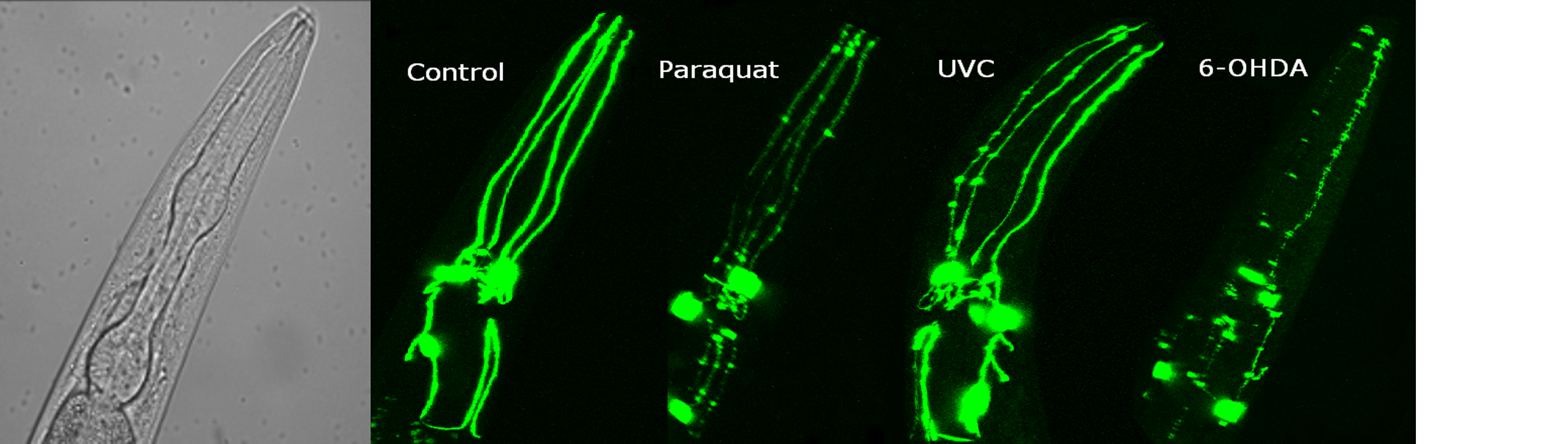

Supplement: S2 Figure — Representative dopaminergic neuron damage after treatment with 180 µM paraquat, 10 J/m2 UVC, and 50 mM 6-hydroxydopamine. Visualized via confocal microscopy. (TIF) [file pone.0114459.s002.tif]

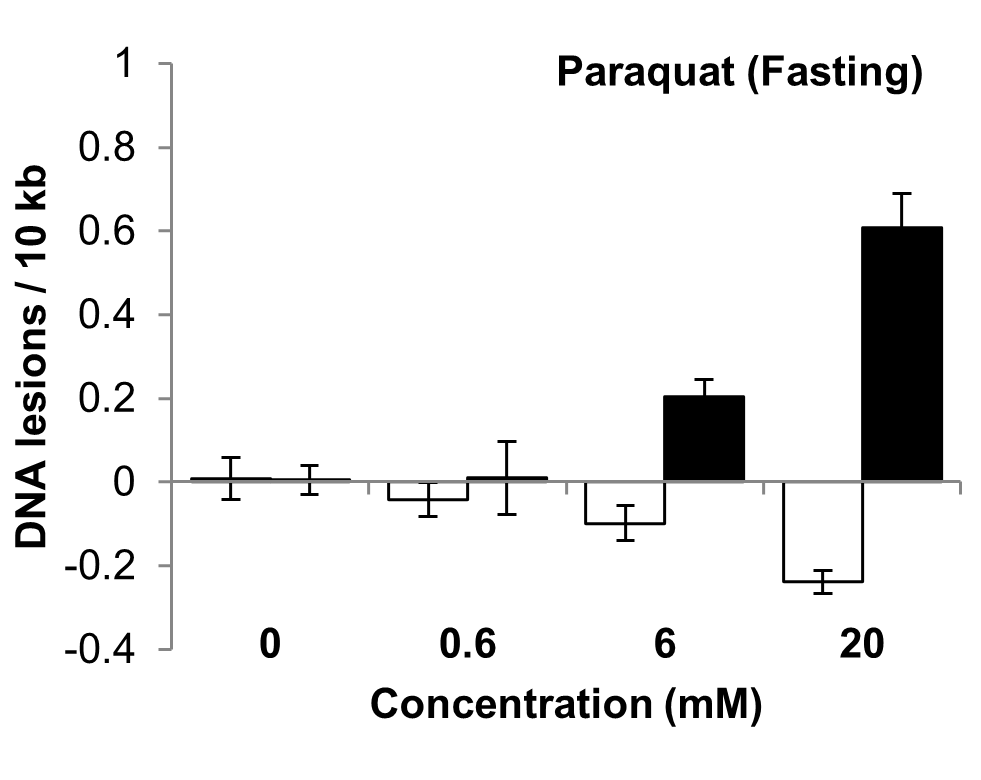

Supplement: S3 Figure — Starvation did not have a protective effect against paraquat exposure. Worms were dosed to 54 µM and 180 µM paraquat after a 48 hr starvation period. (TIF) [file pone.0114459.s003.tif]

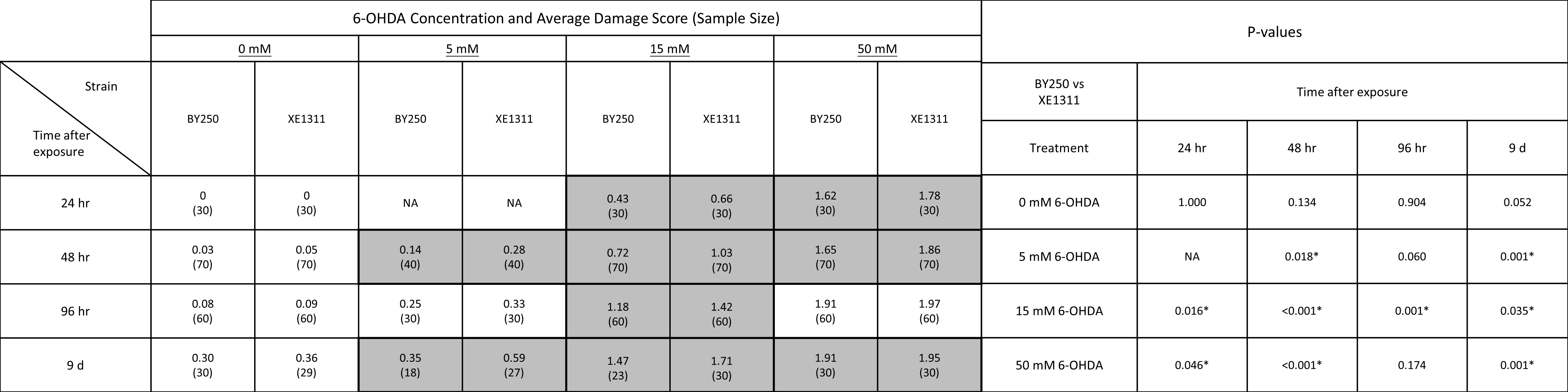

Supplement: S4 Figure — A mutation in the mkk-4 gene required for neuronal regeneration worsens dopaminergic neurodegeneration in 6-OHDA-exposed nematodes. (TIF) [file pone.0114459.s004.tif]

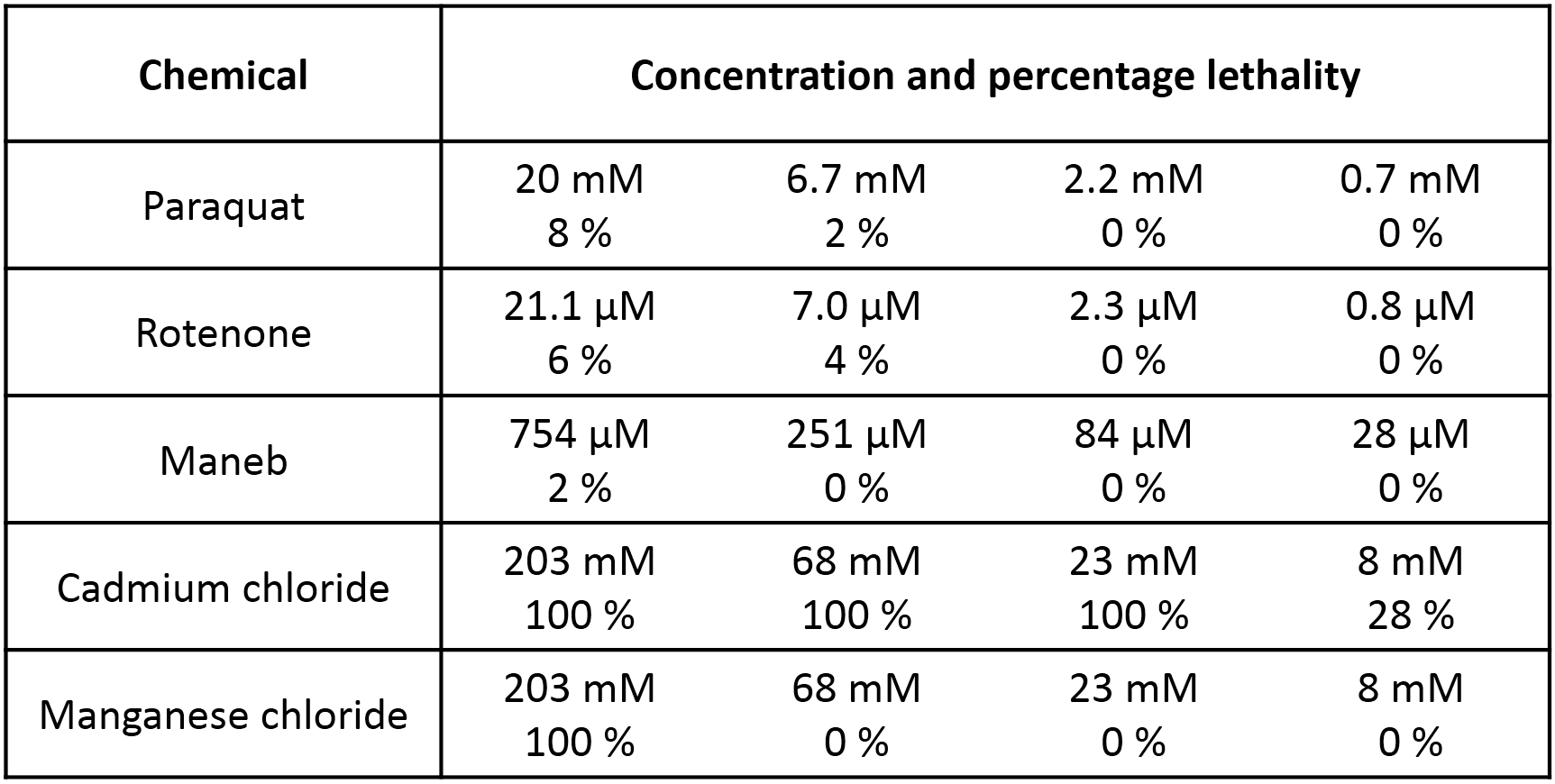

Supplement: S1 Table — Lethality caused by toxins of interest in young adult C. elegans . No lethality was detected in both blank and 1% dimethyl sulfoxide (carrier). n = 100 per chemical per dose. (TIF) [file pone.0114459.s005.tif]

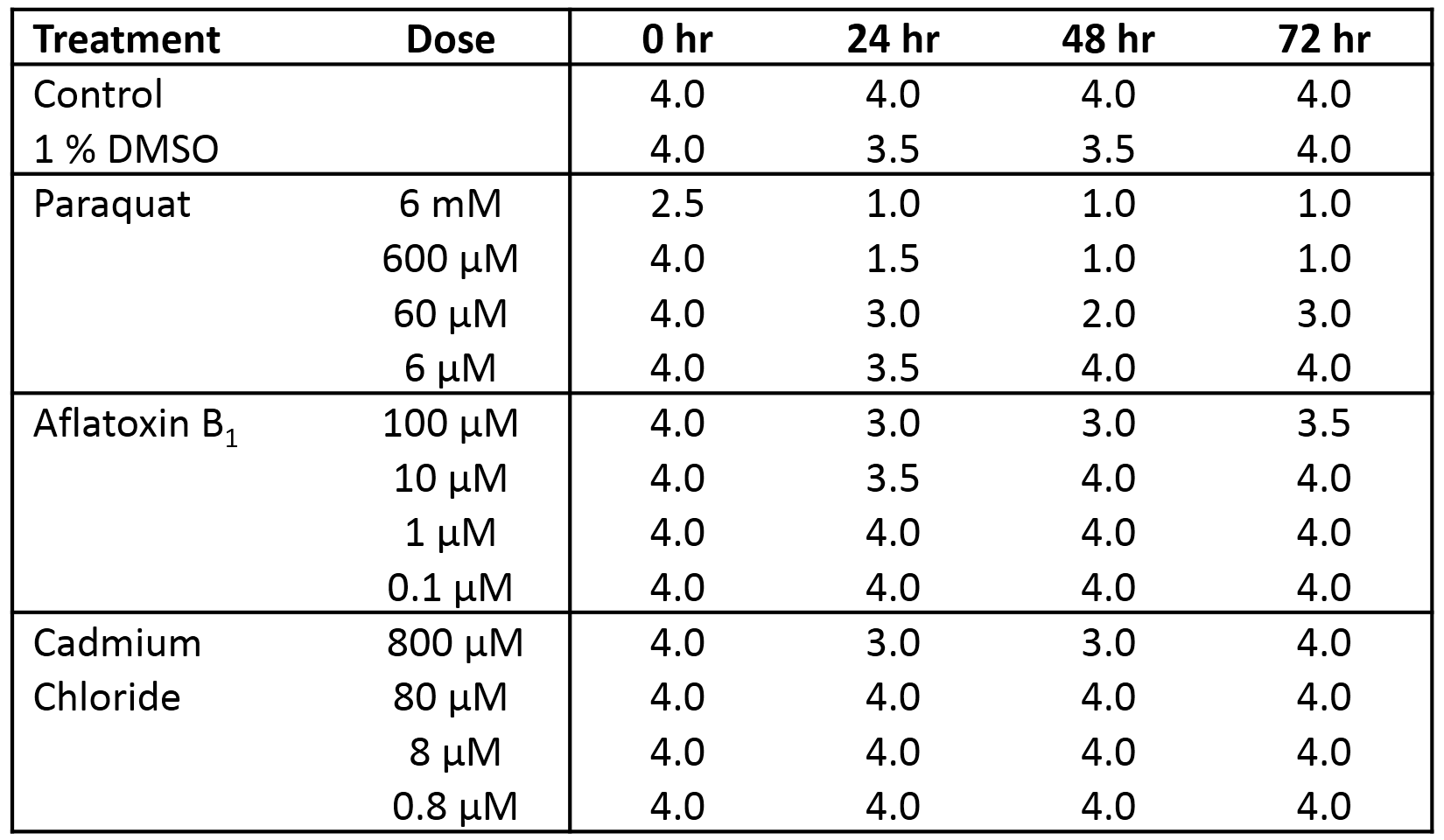

Supplement: S2 Table — Growth inhibition caused by exposure to paraquat, aflatoxin B1 and cadmium chloride in C. elegans . The development of L1 BY250 (n = 4) was compared to control and scored on a 4 point scale: 1: mostly dead and dying; 2: obvious decrease in size and motility; 3: slight decrease in size and motility; 4: similar to control. (TIF) [file pone.0114459.s006.tif]
